# Fulminant Myocarditis with VA-ECMO Support: Clinical Characteristics and Prognosis in a Cohort from a Tertiary Transplant Center

**DOI:** 10.3390/biomedicines13092146

**Published:** 2025-09-03

**Authors:** Borja Guerrero Cervera, Raquel López-Vilella, Ricardo Gimeno Costa, Francisca Pérez Esteban, Manuel Pérez Guillén, Isabel Madrid, Víctor Donoso Trenado, Julia Martínez-Solé, Álvaro Castellanos, Luis Martínez Dolz, Juan Martínez León, Salvador Torregrosa, Luis Almenar-Bonet

**Affiliations:** 1 Cardiology Department, Hospital Universitari i Politècnic La Fe, Avenida Fernando Abril Martorell, Número 106, 46026 Valencia, Spain; cune10@hotmail.com (R.L.-V.); vdonoso@outlook.com (V.D.T.); juliamsole@gmail.com (J.M.-S.); luismartinezdolz@gmail.com (L.M.D.); lualmenar@gmail.com (L.A.-B.); 2 Unit of Heart Failure and Transplant, Hospital Universitari i Politècnic La Fe, 46026 Valencia, Spain; 3 Centro de Investigación Biomédica en Red de Enfermedades Cardiovasculares (CIBERCV), Instituto de Salud Carlos III, 28029 Madrid, Spain; 4 Intensive Care Department, Hospital Universitari i Politècnic La Fe, 46026 Valencia, Spain; gimeno_ric@gva.es (R.G.C.); perez_fraest@gva.es (F.P.E.); castellanos_alv@gva.es (Á.C.); 5 Cardiovascular Surgery Department, Hospital Universitari i Politècnic La Fe, 46026 Valencia, Spain; perez_mgui@gva.es (M.P.G.); martinez_jualeo@gva.es (J.M.L.); torregrosa_sal@gva.es (S.T.)

**Keywords:** fulminant myocarditis, extracorporeal membrane oxygenation, heart transplantation, ventricular function recovery, prognostic biomarkers

## Abstract

**Background/Objectives:** Fulminant myocarditis (FM) is an uncommon but potentially reversible form of myocardial inflammation that can rapidly progress to cardiogenic shock (CS). In patients who are refractory to conventional treatment, venoarterial extracorporeal membrane oxygenation (VA-ECMO) represents an effective life support strategy. However, the factors that determine functional recovery remain uncertain. The primary objective of this study was to characterize patients who recover ventricular function. Secondary objectives included analyzing VA-ECMO-related complications and overall patient survival. **Methods:** This was a retrospective, single-center, observational study including all consecutive patients diagnosed with FM between 2008 and 2025 who were supported with VA-ECMO (n = 22). Clinical, biochemical, echocardiographic, and imaging variables were collected. Patients were classified based on their outcomes as either recovery or death/transplantation. Differential factors potentially affecting myocardial recovery, survival, and complications were analyzed. **Results:** The mean age was 49.7 ± 11 years, with 36% being male. Severe cardiogenic shock was the most common initial presentation (86%), and the average time from symptom onset to hospital admission was 5.7 days. Regarding mechanical support, the non-recovery group required longer ECMO support (328 ± 225 h vs. 188 ± 103 h; *p* = 0.03). The presence of fibrosis on cardiac magnetic resonance imaging (MRI) was associated with a lower probability of recovery (100% vs. 44.4%; *p* = 0.03). Renal failure and vascular complications were more frequent in the non-recovery group, with a significantly higher rate of surgical reintervention (50% vs. 10%; *p* = 0.04). Echocardiography performed before discharge (recovery group) vs. before death/transplant (non-recovery group) showed significant differences in left ventricular ejection fraction (51.1% vs. 29.5%; *p* = 0.04), along with better levels of creatinine, N-terminal pro-B-type natriuretic peptide (NT-proBNP), leukocytes, and C-reactive protein (CRP) in the recovery group. In-hospital survival for the entire cohort was 63.6%, significantly higher in the recovery group (100% vs. 33.3%; *p* < 0.01). One-year survival was 59%, which was also greater among those who recovered (90% vs. 33.3%; *p* = 0.02). **Conclusions**: FM is associated with an acceptable in-hospital survival rate. The presence of myocardial fibrosis on MRI and longer ECMO support duration were observed to be associated with a lower likelihood of cardiac recovery. Patients who recovered showed better ventricular function at discharge, as well as reduced systemic inflammation and renal dysfunction. These findings highlight the importance of early identification of predictors of myocardial recovery to optimize management and therapeutic decision making in this high-risk population.

## 1. Introduction

Myocarditis is an inflammatory disease of the myocardium with a wide range of clinical presentations, from asymptomatic cases to severe forms like cardiogenic shock. Its global incidence is estimated at 1–10 per 100,000 per year, though autopsy studies of sudden cardiac death in young adults show histological myocarditis in 8–12% of cases [1].

Fulminant myocarditis (FM) is an uncommon and severe form of acute myocarditis, accounting for approximately 10% of all cases. It typically presents as a critical condition requiring intensive care management. In some cases, inotropic support and conventional therapies are sufficient, but in others, mechanical circulatory support is required to stabilize the patient while assessing the potential for myocardial recovery or candidacy for heart transplantation (HTx) [2].

Venoarterial extracorporeal membrane oxygenation (VA-ECMO) has become a key therapeutic tool in the management of FM, particularly in patients with CS refractory to standard treatment. Its timely initiation can be decisive for survival and functional myocardial recovery [2,3]. Some studies report that up to 60–70% of patients with FM treated with VA-ECMO survive with complete ventricular function recovery. However, most of these series are small due to the low prevalence of this condition [4].

To date, it remains unclear which clinical factors differ between patients who recover myocardial function and those who either die or require heart transplantation. The hypothesis of this study was that a detailed clinical characterization of FM patients supported with VA-ECMO could help identify those more likely to experience ventricular recovery and favorable clinical outcomes.

Therefore, the primary objective of this study was to share our center’s experience in the clinical characterization of patients diagnosed with FM and supported with VA-ECMO, in order to evaluate parameters associated with ventricular function recovery. Additionally, we aimed to describe ECMO-related complications and assess overall patient survival.

## 2. Materials and Methods

This was a retrospective observational study conducted between February 2008 and January 2025. Inclusion criteria were as follows: all adult patients (≥18 years) admitted to the intensive care unit (ICU) of a tertiary referral center between February 2008 and January 2025 with a diagnosis of FM according to ESC/AHA definitions [5,6], requiring VA-ECMO support for refractory cardiogenic shock. FM was defined by acute onset of severe ventricular dysfunction (LVEF ≤ 30% and/or severe right ventricular dysfunction), rapid hemodynamic deterioration, and absence of significant coronary artery disease confirmed by urgent coronary angiography when possible or available. Echocardiographic parameters considered included fractional shortening ≤ 15% and/or tricuspid annular plane systolic excursion (TAPSE) < 16 mm. Patients with incomplete clinical data were excluded. A total of 22 patients were included, of whom 10 recovered ventricular function, and 12 did not (4 underwent heart transplantation and 8 died).

Acute kidney injury (AKI) was defined according to Kidney Disease: Improving Global Outcomes (KDIGO) criteria, with renal failure defined as KDIGO stage 3 or need for renal replacement therapy [7]. Institutional criteria for ECMO initiation included refractory cardiogenic shock (cardiac index < 2.0 L/min/m^2^ despite maximal inotropic support), persistent hyperlactatemia > 4 mmol/L, or progressive multiorgan failure; escalation to additional mechanical circulatory support was performed in cases of left ventricular distension or refractory pulmonary edema.

The diagnosis of FM was based on the sudden onset of severe ventricular dysfunction in previously healthy individuals, with rapid progression to cardiogenic shock requiring inotropic and/or mechanical support. Coronary artery disease was ruled out in all cases via coronary angiography. The diagnosis was supported by acute clinical presentation, severe ventricular dysfunction on echocardiography (with normal or reduced ventricular dimensions), significantly elevated cardiac injury and inflammatory biomarkers, and exclusion of other causes of shock [8,9]. All included patients required VA-ECMO as life support due to refractory hemodynamic failure.

Demographic, clinical, and diagnostic data at admission were collected, including hemodynamic status, echocardiographic findings, cardiac magnetic resonance imaging (MRI) [if available], inflammatory markers, and cardiac injury biomarkers. Initial therapeutic management was analyzed, including the use of inotropic support, immunomodulatory therapy, and mechanical circulatory assistance (VA-ECMO [Cardiohelp System, Getinge, Gothenburg, Sweden]), Impella® (Abiomed, Danvers, MA, USA), intra-aortic balloon pump, etc.). Time to ventricular function recovery, duration of mechanical support, associated complications, and total length of ICU and hospital stay were also documented.

Infectious complications included any clinically suspected or microbiologically confirmed infection associated with signs or symptoms and/or elevated inflammatory markers that prompted initiation or escalation of antibiotic therapy. If infection-induced organ dysfunction was present, it was defined as sepsis. Pulmonary embolism (PE) was confirmed by computed tomography (CT) and/or pulmonary angiography, and deep vein thrombosis (DVT) was diagnosed by Doppler ultrasound. Renal failure was defined as the need for renal replacement therapy (RRT), including ultrafiltration and/or dialysis, at any time during hospitalization. Vascular complications included any issue related to vascular access for mechanical support. Reoperations were defined as unplanned surgeries performed during the same hospital stay, either due to complications related to mechanical support implantation or new clinical conditions requiring surgical management. Tracheostomy was considered a complication when performed due to prolonged inability to wean from invasive mechanical ventilation or for airway protection. Severe neuromyopathy was defined as generalized muscle weakness significantly interfering with the functional recovery process, diagnosed through compatible clinical examination and/or neurophysiological studies.

This study was conducted in accordance with the Declaration of Helsinki. The research project was approved by the Biomedical Research Ethics Committee of the University and Polytechnic Hospital La Fe, Valencia, Spain.

### Statistical Analysis

Descriptive analysis was performed. Continuous variables were expressed as mean ± standard deviation or median with interquartile range, depending on data distribution. Categorical variables were expressed as frequencies and percentages. Group comparisons were performed using Student’s *t*-test for independent samples or the Mann–Whitney U test for continuous variables, and Chi-square or Fisher’s exact test was performed for categorical variables. Time-to-event analysis was conducted using Kaplan–Meier survival curves and compared using the log-rank test. A *p*-value < 0.05 was considered statistically significant. In the Kaplan–Meier survival analysis, both events (death and transplantation) were considered as endpoints because both represent a failure of ventricular recovery and initial treatment, and separating them would have resulted in very small subgroups, limiting statistical power. Statistical analysis was performed using IBM SPSS Statistics Version 27^®^ and Stata^®^ Statistics/Data Analysis Version 16.1. Graphs were created using SPSS and PowerPoint.

## 3. Results

A total of 22 patients diagnosed with FM requiring VA-ECMO support were included. The mean age was 49.7 ± 12 years, and 36% were male. No significant differences were observed between the recovery and non-recovery groups regarding age, sex, or cardiovascular risk factors. Similarly, there were no significant differences in the time from symptom onset to ICU admission (Table 1).

In terms of clinical presentation, cardiogenic shock was the predominant reason for ICU admission in both groups, which was present in 100% of non-recovery patients and in 70% of those who recovered. However, the duration of ECMO support was significantly longer in the non-recovery group (328 ± 225 h vs. 188 ± 103 h; *p* = 0.03). All patients received vasoactive support (norepinephrine and dobutamine). The use of left ventricular assist devices (mainly Impella^®^ and intra-aortic balloon pump) was more frequent in the non-recovery group, though not statistically significant (*p* = 0.34).

MRI revealed that myocardial fibrosis was significantly more frequent in the non-recovery group (100% vs. 44.4%; *p* = 0.03), although fewer patients in this group underwent MRI. No significant differences were found in the presence of myocardial edema. Endomyocardial biopsy was performed in only two patients in the entire cohort (Table 2).

At admission, no significant differences in biomarker levels were observed between groups (Table 3). Initial left ventricular ejection fraction (LVEF) values were severely reduced in both groups, without significant differences (17.4 ± 7.6% in recovery vs. 12.6 ± 6.7% in non-recovery; *p* = 0.195) (Table 3). However, at discharge (recovery group) or prior to death/transplantation (non-recovery group), a significantly higher LVEF was observed in recovered patients (51.1 ± 13.1% vs. 29.5 ± 23.2%; *p* = 0.04), along with lower right ventricular dysfunction (*p* = 0.01). Additionally, recovered patients showed better renal function (creatinine 0.7 ± 0.2 mg/dL vs. 1.2 ± 0.6 mg/dL; *p* = 0.01) and significantly lower levels of NT-proBNP (1981 ± 2314 pg/mL vs. 15,687 ± 14,974 pg/mL; *p* < 0.01), leukocyte count (8828 ± 2538 vs. 18,485 ± 11,009; *p* = 0.01), and C-reactive protein (CRP) (23 ± 18 mg/L vs. 98 ± 96 mg/L; *p* = 0.02) (Table 4).

**Figure 1:**
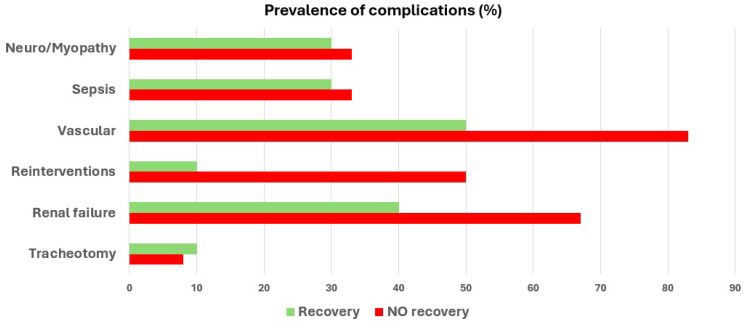
Complications during ICU stay.

Complications were more frequent in the non-recovery group, including renal dysfunction (67% vs. 40%; *p* = 0.19) and vascular complications (83% vs. 50%; *p* = 0.07), with a significantly higher rate of surgical reoperations (50% vs. 10%; *p* = 0.04). Rates of sepsis and tracheostomy were similar between groups (Table 5) (Figure 1).

Four patients underwent HTx, all from the non-recovery group. The mean ICU stay was 21 ± 16 days, with no significant differences between groups. Time to clinical recovery or death was also similar (Table 5).

**Figure 2:**
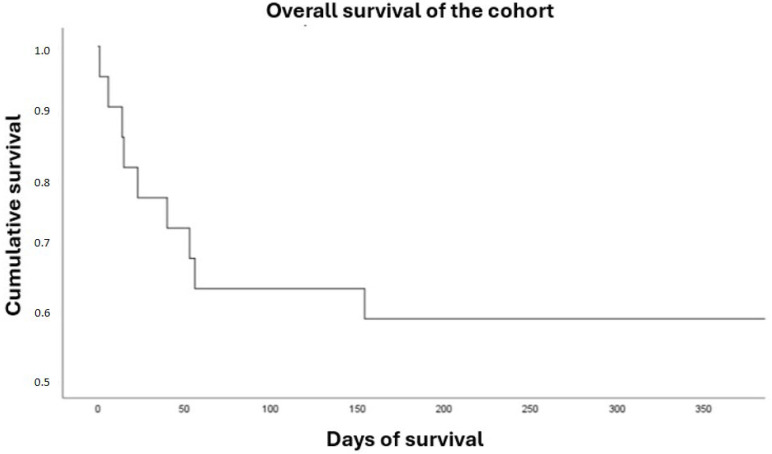
Overall survival.

**Figure 3:**
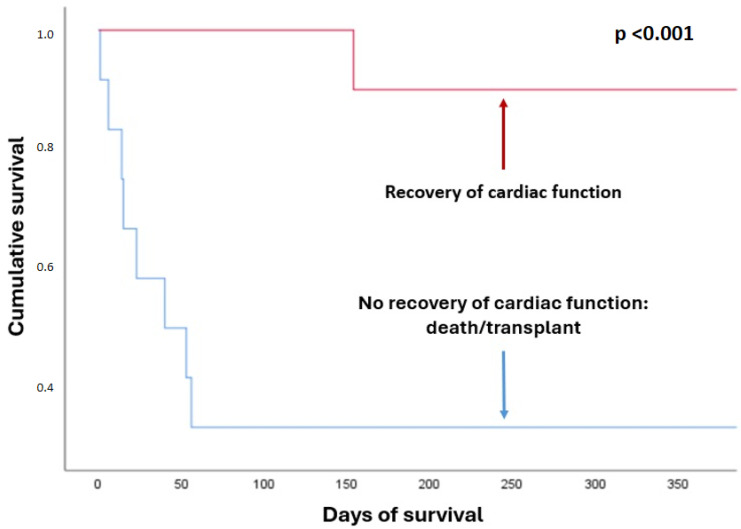
Survival by outcome group.

Overall in-hospital survival for the entire cohort was 63.6%, being significantly higher in the recovery group (100% vs. 33.3%; *p* < 0.01). One-year survival was 59%, which was also higher in the recovery group (90% vs. 33.3%; *p* = 0.02) (Table 5) (Figure 2 and Figure 3). In the Kaplan–Meier survival analysis, both death and heart transplantation were analyzed as equivalent endpoints, since both outcomes represent failure of ventricular recovery and initial treatment.

## 4. Discussion

FM is an uncommon but potentially reversible form of myocardial inflammation that may rapidly progress to CS and multiorgan failure. In such cases, VA-ECMO has become a first-line life support strategy [1,3]. This study presents the experience of a tertiary care center in managing 22 patients with FM who received VA-ECMO. We observed that CS was the most frequent clinical presentation and that longer ECMO support duration was associated with a lack of cardiac recovery. Approximately half of the patients achieved full recovery of left ventricular function, with significantly higher ejection fractions at discharge compared to those who required transplantation or died. The presence of myocardial fibrosis on cardiac MRI was significantly associated with lack of recovery. Conversely, patients with favorable outcomes exhibited less right ventricular dysfunction, lower systemic inflammation, and better renal function at discharge compared to non-recovered patients at the time of death or transplantation.

In our cohort, the average time from symptom onset to hospital admission was 5.7 days, with no significant differences between groups. This reflects the abrupt and aggressive nature of FM, which typically evolves within hours to a few days from nonspecific symptoms to hemodynamic collapse [8]. While previous studies have reported a higher prevalence of FM in young males [10], our series did not demonstrate significant sex-related differences. Males accounted for only 36% of the cohort, which may reflect sampling bias or a higher prevalence of non-viral etiologies—such as autoimmune forms—among females. Other series have also shown no clear sex predominance or even a higher frequency in women [1,11].

CS was the dominant clinical manifestation, observed in 86% of cases, aligning with previous studies reporting CS as the most common presentation among FM patients requiring ECMO [12]. Although less frequent in our series, arrhythmic presentations can be the initial form in up to 10–15% of cases, particularly in autoimmune or post-infectious settings [13]. Most cases in our study were classified as idiopathic, followed by viral and autoimmune etiologies—consistent with the diagnostic challenges of FM and similar to patterns reported in other cohorts [1,9]. Viral forms remain the most common, although fulminant autoimmune and hypersensitivity-related myocarditis have been increasingly described and may carry a better prognosis when identified and treated early [1,14].

ECMO duration was significantly longer in patients who did not recover, suggesting that prolonged support may be indicative of more severe disease or a lower likelihood of myocardial recovery. This finding is consistent with studies advocating early ECMO initiation and the use of low-flow settings to reduce complications and improve outcomes [15,16]. Chow HW et al. reported that ECMO durations beyond 7 days are associated with lower chances of recovery without transplantation, especially in the absence of echocardiographic improvement within the first 3–5 days [15].

Use of left ventricular assist devices (LVADs), such as Impella^®^ or intra-aortic balloon pump, was more frequent in the non-recovery group, though not statistically significant. The need for biventricular support or left ventricular unloading is considered a marker of disease severity and may reflect more persistent or severe dysfunction [17]. In specialized centers, combining VA-ECMO with Impella^®^ or TandemHeart has shown benefits in improving survival and reducing left ventricular overload, although these strategies are not yet standardized [18].

Cardiac MRI played a key role in diagnosis and prognostic stratification. In our series, the presence of myocardial fibrosis was significantly more common in the non-recovery group. This supports existing evidence linking fibrosis to irreversible myocardial injury and a lower likelihood of functional recovery [19]. Conversely, myocardial edema—observed in both groups—did not correlate with prognosis. It should be noted that cardiac MRI was not performed in all patients, particularly in the non-recovery group, where clinical instability and the technical complexity of transferring patients on VA-ECMO to the MRI suite frequently precluded the procedure; this reflects the clinical reality of managing critically ill patients on VA-ECMO. Additionally, the variable timing of cardiac MRI relative to ECMO initiation represents a potential source of selection bias, as only clinically stable patients were able to undergo the procedure. This variability limits the generalizability of our findings. Endomyocardial biopsy was performed in only a few patients due to procedural risk and logistical challenges in unstable patients on full circulatory support, and did not yield significant findings, likely due to its limited diagnostic value in acute stages and the procedural risks in hemodynamically unstable patients [20].

Recovered patients exhibited less right ventricular dysfunction and better markers of renal function, systemic inflammation, and myocardial stress. These findings are in line with prior studies highlighting the prognostic importance of right ventricular function in critically ill patients [21]. Right ventricular dysfunction, often underestimated, may indicate severe hemodynamic compromise and systemic congestion, hindering myocardial recovery and favoring multiorgan dysfunction [8,21]. Likewise, improved renal function and lower levels of inflammatory markers such as CRP and myocardial stress markers like NT-proBNP in recovered patients may reflect a less aggressive systemic state and a more favorable environment for functional recovery [22,23].

ECMO-related complications were common in our cohort, reflecting the high complexity of these cases. Acute kidney injury often required renal replacement therapy, and vascular complications—including limb ischemia, bleeding at cannulation sites, and retroperitoneal hematomas—were prevalent. These complications not only increase morbidity and mortality but also prolong ICU stay and hinder early mobilization and rehabilitation. Surgical reintervention rates were significantly higher in the non-recovery group, suggesting that complications may act both as markers and perpetuators of multiorgan dysfunction. This aligns with systematic reviews and observational studies identifying hemorrhagic, infectious, neurological, and vascular complications as independent predictors of mortality in VA-ECMO patients [24,25]. Acute kidney injury, in particular, may affect over 70% of patients in some series and stands out as one of the strongest prognostic indicators of adverse outcomes [26]. Similarly, distal ischemia due to femoral cannulation and the need for vascular revision surgery or fasciotomy have been linked to worse outcomes, including a higher risk of amputation and sepsis [27].

From a functional standpoint, 45% of patients in our series achieved left ventricular recovery without the need for transplantation—comparable to other reports, which estimate recovery rates between 40% and 60%, depending on the population studied [8,28]. Among survivors, left ventricular ejection fraction significantly improved from 17.4% to 51.1% at discharge. These findings support the potential reversibility of FM when timely and appropriate mechanical support is provided. This pattern of rapid recovery in the absence of fibrosis is characteristic of FM and contrasts with the more progressive course of other cardiomyopathies [8,29].

Our results are consistent with the international literature supporting the effectiveness of VA-ECMO in FM. In-hospital survival was 63.6%, comparable to the 59% reported by Chow HW et al. [15] and slightly lower than the 71.9% observed by Lorusso et al. [2]. Our recovery rate without transplantation was 45%. A systematic review of 170 cases by Cheng et al. reported recovery in over 66% of patients treated with ECMO [4]. These discrepancies may be explained by our limited sample size and the higher baseline severity and complication rates in our cohort.

The diagnosis and risk stratification of FM without endomyocardial biopsy remains challenging, and early ECMO support may be lifesaving while the underlying etiology is clarified, showing higher survival rates compared to other causes of cardiogenic shock and serving as a bridge to recovery or advanced therapies. Furthermore, targeted immunosuppressive strategies with antithymocyte globulin (ATG) or OKT3 (muromonab-CD3) have been reported—mainly in case series and reports—as potential adjunctive therapies in giant cell myocarditis and other fulminant presentations, especially when combined with ECMO and corticosteroids [30,31]. Finally, ECMO induces both inflammatory activation and secondary immunosuppression, increasing susceptibility to nosocomial infections; therefore, close infection monitoring, strict aseptic techniques, and context-specific peri-cannulation antibiotic prophylaxis should be considered, in line with current guidelines that discourage routine prophylaxis and emphasize antimicrobial stewardship [32].

This study has several limitations. The small sample size reflects the low prevalence of FM and the specific indication for VA-ECMO support, which may limit statistical power. Its retrospective, single-center nature introduces potential selection bias and limits generalizability, so the results should be interpreted with caution. In the survival analysis, both death and heart transplantation were treated as equivalent endpoints. This choice was driven by the limited sample size, since analyzing the two outcomes separately would have resulted in very small subgroups and insufficient statistical power. This approach may introduce grouping bias. The long study period (2008–2025) may also imply potential era-related effects; however, stratified analyses were not feasible due to sample size constraints. Not all patients underwent cardiac MRI due to their critical status, and endomyocardial biopsy was not routinely performed; thus, histopathological data were not analyzed. Finally, another limitation is the variability in the timing of cardiac MRI in relation to ECMO initiation, since only patients who were clinically stable could undergo the procedure. Moreover, the lack of systematic and early MRI limits its applicability as a prognostic tool in acute decision making, since results may not always be available at the time when critical therapeutic decisions are required. Nonetheless, this study has notable strengths. It represents one of the largest single-center series of FM patients treated with VA-ECMO in our national context. The diagnostic consistency and prolonged follow-up allow for robust conclusions regarding clinical evolution and recovery factors. The detailed characterization—including clinical, echocardiographic, laboratory, and advanced imaging parameters—offers a comprehensive risk profile and facilitates early identification of recovery predictors. Furthermore, the systematic analysis of ECMO-related complications, often underreported, highlights the complexity of managing these critically ill patients. Finally, the fact that all patients were treated at a high-volume tertiary care center with established expertise in advanced circulatory support ensures uniformity in management protocols, representing an additional methodological strength. These findings may serve as a foundation for future multicenter studies and meta-analyses with greater statistical power.

## 5. Conclusions

FM requiring VA-ECMO support is associated with an acceptable in-hospital survival rate. A significant proportion of patients achieve ventricular function recovery without the need for transplantation, although complications are frequent. The presence of myocardial fibrosis on cardiac MRI and a longer duration of VA-ECMO support were observed to be associated with a lower likelihood of cardiac recovery. Patients who recovered showed better ventricular function at discharge, as well as lower levels of systemic inflammation and renal dysfunction. These findings highlight the importance of identifying early predictors of myocardial recovery in order to optimize management and therapeutic decision making in this high-risk population.

## Figures and Tables

**Table 1:** Baseline characteristics.

	Recovery (n: 10)	No Recovery (n: 12)	*p*	Total (n = 22)
Age (years) *	47 ± 14	52 ± 8	0.30	49.7 ± 11
Male (n, %)	5 (50)	3 (25)	0.37	8 (36.4)
Comorbidities (n, %)				
HT	1 (10)	2 (16.6)	0.48	3 (13.6)
DL	1 (10)	1 (8.3)	0.75	2 (9.1)
DM	0 (0)	1 (8.3)	0.95	1 (4.5)
Active smoking	1 (10)	1 (8.3)	0.95	2 (9.1)
CKD	1 (10)	2 (16.6)	0.67	3 (13.6)
Weight (kg) *	56 ± 11	61 ± 13	0.40	58.7 ± 12.1
Height (cm) *	173 ± 13	173 ± 10	0.97	173 ± 11.4
Previous CV disease	1 (10)	3 (25)	0.59	4 (18.2)
Time from symptom onset to ICU admission (days) *	5.2 ± 5.4	6.1 ± 3.7	0.56	5.7 ± 4.5

* Kolmogorov–Smirnov > 0.05, mean ± standard deviation. Kolmogorov–Smirnov < 0.05, median ± interquartile range. (n, %): number and percentage. Abbreviations: CKD: chronic kidney disease; CV: cardiovascular; DL: dyslipidaemia; DM: diabetes mellitus; HT: hypertension; ICU: intensive care unit.

**Table 2:** Clinical characteristics of patients with fulminant acute myocarditis.

	Recovery (n = 10)	No Recovery (n = 12)	*p*	Total (n = 22)
Clinical presentation (n, %): Cardiogenic shock (low output) Arrhythmic Other	7 (70) 2 (20) 1 (10)	12 (100) 0 (0) 0 (0)	0.06	19 (86.4) 2 (9.1) 1 (4.5)
INTERMACS level (n, %) I II	1 (10) 9 (90)	4 (33) 8 (67)	0.21	5 (22.7) 17 (77.3)
ECMO hours *	188 ± 103	328 ± 225	0.03	267 ± 281
Intubation required (n, %)	8 (80)	11 (91.6)	0.54	19 (86.4)
Duration of invasive mechanical ventilation (hours) *	162 ± 68	210 ± 103	0.11	189 ± 90
Left ventricular assist device (LVAD) (n, %): Impella Intra-aortic balloon pump	3 (30) 0 (0) 3 (100)	6 (50) 3 (50) 3 (50)	0.34	9 (40.9) 3 (13.6) 6 (27.2)
LVAD duration (hours)	197 ± 175	245 ± 147	0.701	223 ± 161
Catheterization	5 (50)	10 (83.3)	0.09	15 (68.2)
Cardiac MRI (n, %): Fibrosis Edema	9 (90) 4 (44.4) 8 (88.8)	6 (50) 6 (100) 3 (50)	0.12 0.03 0.28	15 (68.18) 10 (45.45) 11 (50)
Endomyocardial biopsy (n,%)	1 (10)	1 (8.3)	0.98	2 (9.1)
Drugs (n, %): NorepinephrineDobutamine Corticosteroids Immunosuppressants	10 (100) 10 (100) 2 (20) 0 (0)	12 (100) 12 (100) 2 (16.6) 0 (0)	0.99	22 (100) 22 (100) 4 (18.2) 0 (0)
Etiologic diagnosis (n, %): Viral Autoimmune Idiopathic	2 (20) 2 (20) 6 (60)	2 (16.7) 0 (0) 10 (83.3)	0.56	4 (18.2) 2 (9.1) 16 (72.7)

* Kolmogorov–Smirnov > 0.05, mean ± standard deviation. Kolmogorov–Smirnov < 0.05, median ± interquartile range. (n, %): number and percentage. Abbreviations: ECMO: extracorporeal membrane oxygenation; LVAD: left ventricular assist device; MRI: magnetic resonance imaging.

**Table 3:** Echocardiographic and laboratory characteristics of patients with fulminant acute myocarditis.

	Recovery (n = 10)	No Recovery (n = 12)	*p*	Total (n = 22)
Echocardiographic parameters at admission: LVEF (%) * LVEF with severe dysfunction (n, %) RV severe dysfunction (n, %) PASP *	17.4 ± 7.6 10 (100) 5 (50) 30.6 ± 3.4	12.6 ± 6.7 12 (100) 8 (66.6) 41.2 ± 4.7	0.19 0.98 0.54 0.06	14.8 ± 7.3 22 (100) 13 (591) 36.4 ± 6.7
Laboratory parameters at admission: * Creatinine (mg/dL)GFR (mL/min) Bilirubin (mg/dL) AST (GOT) (U/L) ALT (GPT) (U/L) Lactate (mmol/L) NT-proBNP (pg/mL) Troponin (pg/mL) Leukocytes (cells/µL) C-reactive protein (mg/L)	1.6 ± 0.7 56.6 ± 25.6 1.7 ± 1.3 1471 ± 3192 1902 ± 3789 5.5 ± 3.9 20,351 ± 12,652 3337 ± 3371 17,487 ± 8364 138 ± 136	1.4 ± 0.7 60.3 ± 36.8 1.8 ± 1.4 3084 ± 4147 1801 ± 2157 8.8 ± 6.1 23,711 ± 11,473 5993 ± 6851 14,048 ± 5407 111 ± 108	0.58 0.82 0.87 0.32 0.93 0.20 0.52 0.27 0.25 0.59	1.5 ± 0.7 58.6 ± 31.6 1.75 ± 1.3 2325 ± 3847 1847 ± 2942 7.3 ± 5.4 22,130 ± 12,011 4789 ± 5555 15,687 ± 6986 123 ± 121

* Kolmogorov–Smirnov > 0.05, mean ± standard deviation. Kolmogorov–Smirnov < 0.05, median ± interquartile range. (n, %): number and percentage. Abbreviations: ALT (GPT), alanine aminotransferase; AST (GOT), aspartate aminotransferase; GFR: glomerular filtration rate; LVEF: left ventricle ejection fraction; NT-proBNP, N-terminal pro-B-type natriuretic peptide; PASP: pulmonary artery systolic pressure; RV: right ventricle.

**Table 4:** Echocardiographic and laboratory characteristics at discharge.

	Recovery (n = 10)	No Recovery (n = 12)	*p*	Total (n = 22)
Echocardiographic parameters *#: LVEF (%) * LVEF with severe dysfunction (n,%) RV severe dysfunction (n, %)	51.1 ± 13.1 1 (10) 0 (0)	29.5 ± 23.2 8 (67) 7 (58.3)	0.04 <0.01 0.01	39.3 ± 20.4 9 (40.9) 7 (31.8)
Laboratory parameters at discharge: * Creatinine (mg/dL) GFR (mL/min) Bilirubin (mg/dL) AST (GOT) (U/L) ALT (GPT) (U/L) Lactate (mmol/L) NT-proBNP (pg/mL) Troponin (pg/mL) Leukocytes (cells/µL) C-reactive protein (mg/L)	0.7 ± 0.2 104 ± 17 0.6 ± 0.2 34.1 ± 7.7 52 ± 34 0.8 ± 0.2 1981 ± 2314 190 ± 384 8828 ± 2538 23 ± 18	1.2 ± 0.6 69 ± 36 2.1 ± 2.4 1361 ± 2804 919 ± 2068 4.3 ± 5.8 15,687 ± 14,974 1741 ± 3017 18,485 ± 11,009 98 ± 96	0.01 0.02 0.07 0.12 0.17 0.08 <0.01 0.10 0.01 0.02	1.0 ± 0.5 85.6 ± 31.6 1.4 ± 1.9 748 ± 1943 531 ± 1443 2.7 ± 4.4 9266 ± 11,465 1034 ± 2043 14,188 ± 8370 63 ± 74

* Kolmogorov–Smirnov > 0.05, mean ± standard deviation. Kolmogorov–Smirnov < 0.05, median ± interquartile range. # Echocardiography prior to discharge (recovery group) vs. echocardiography prior to death/transplantation (non-recovery group). (n, %): number and percentage. Abbreviations: ALT (GPT), alanine aminotransferase; AST (GOT), aspartate aminotransferase; GFR: glomerular filtration rate; LVEF: left ventricle ejection fraction; NT-proBNP, N-terminal pro-B-type natriuretic peptide; PASP: pulmonary artery systolic pressure; RV: right ventricle.

**Table 5:** Evolution.

	Recovery (n = 10)	No Recovery (n = 12)	*p*	Total (n = 22)
Heart transplant (n, %)	0 (0)	4 (33.3)	<0.01	4 (18.2)
Days from inclusion in heart transplant waiting list to transplant *	-	5.3 ± 5.2		5.3 ± 5.2
ICU stay (days) *	18.4 ± 11.9	23.2 ± 18.8	0.24	21.1 ± 16.1
Days to recovery (discharge)/death *	33.4 ± 17.7	35.9 ± 28.9	0.31	34.8 ± 24.1
Complications (n, %): Renal failure Vascular complications Reoperations DVT/PE Tracheotomy Severe neuromyopathy Sepsis	4 (40) 5 (50) 1 (10) 0 (0) 1 (10) 3 (30) 3 (30)	8 (67) 10 (83.3) 6 (50) 0 (0) 1 (8) 4 (33) 4 (33)	0.19 0.07 0.04 0.99 0.71 0.61 0.61	12 (54.5) 15 (68.2) 7 (31.8) 0 (0) 2 (9.1) 7 (31.8) 7 (31.8)
In-hospital survival (n, %)	10 (100)	4 (33.3)	<0.01	14 (63.6)
1-year survival (n, %)	9 (90)	4 (33.3)	0.02	13 (59)

* Kolmogorov–Smirnov > 0.05, mean ± standard deviation. Kolmogorov–Smirnov < 0.05, median ± interquartile range. (n, %): number and percentage. Abbreviations: DVT: deep vein thrombosis; HF: heart failure; ICU: intensive care unit; PE: pulmonary embolism.

## Data Availability

The dataset is available upon request to the authors.
